# Supplementary material for: Beech cupules as keystone structures for soil fauna
Source: PeerJ. 2016 Oct 19;4:e2562. doi: 10.7717/peerj.2562 (PMC5075700; doi:10.7717/peerj.2562)
Supplement: Supplemental Information 1 — Appendix 1, 2 and 3 (Beech cupules as keystone structures for soil fauna) [file peerj-04-2562-s001.doc]

**Supplemental  Information  - Appendix 1**

**
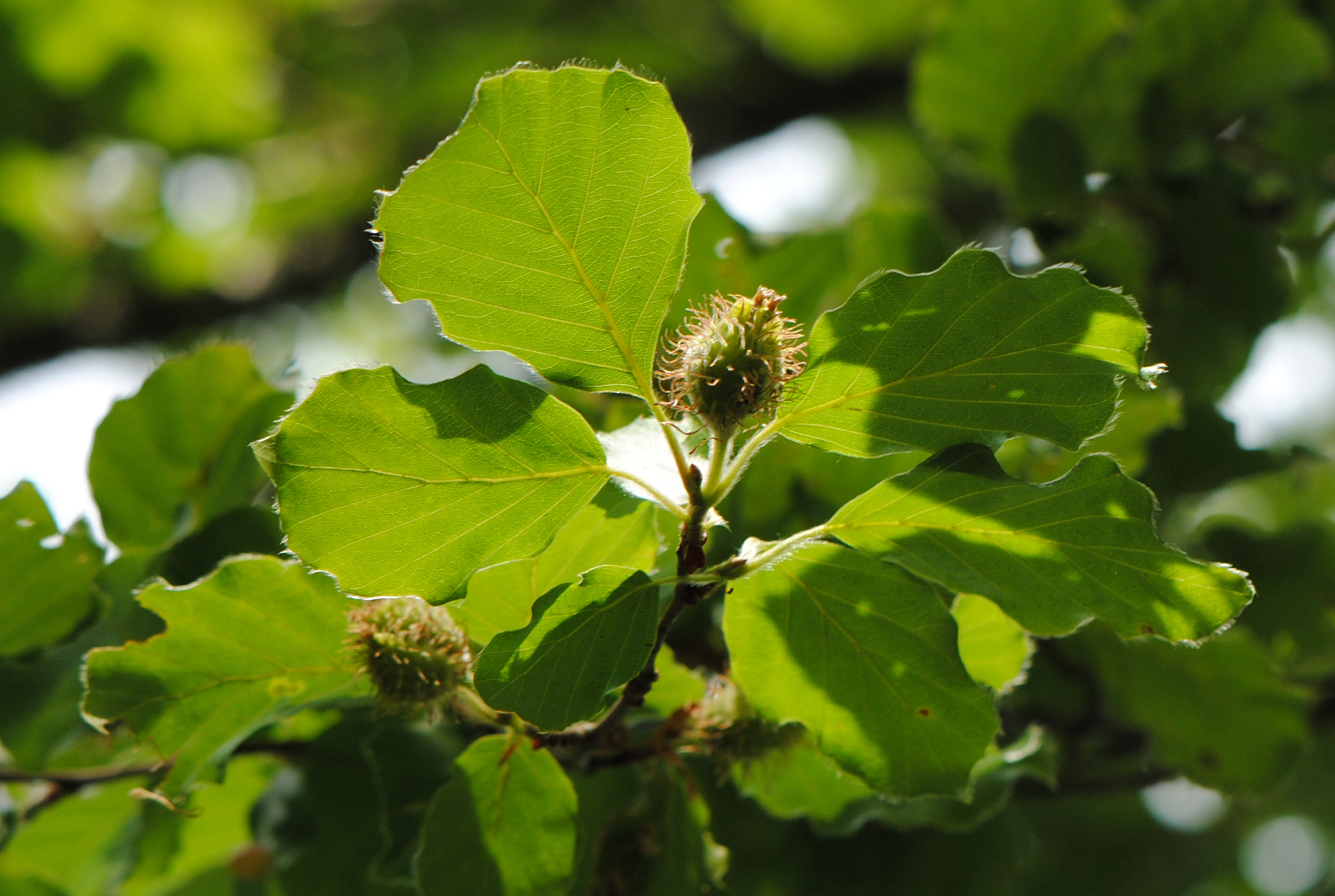
**

**
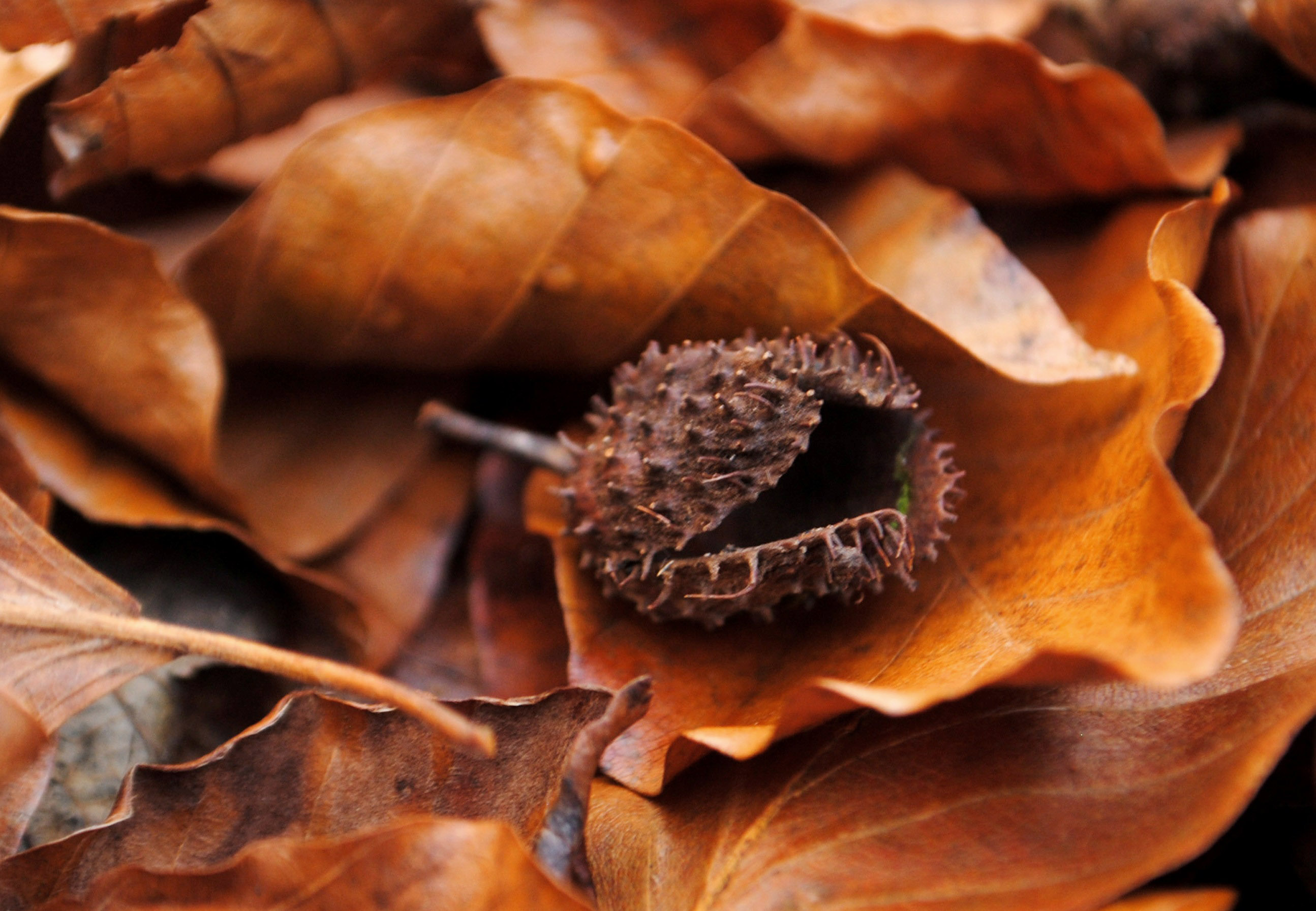
**

**Figure S1.** Beech cupules before ripening in late spring (left) and dropped on the forest floor after ripening (right). Photo credit: Nereida Melguizo-Ruiz.

**
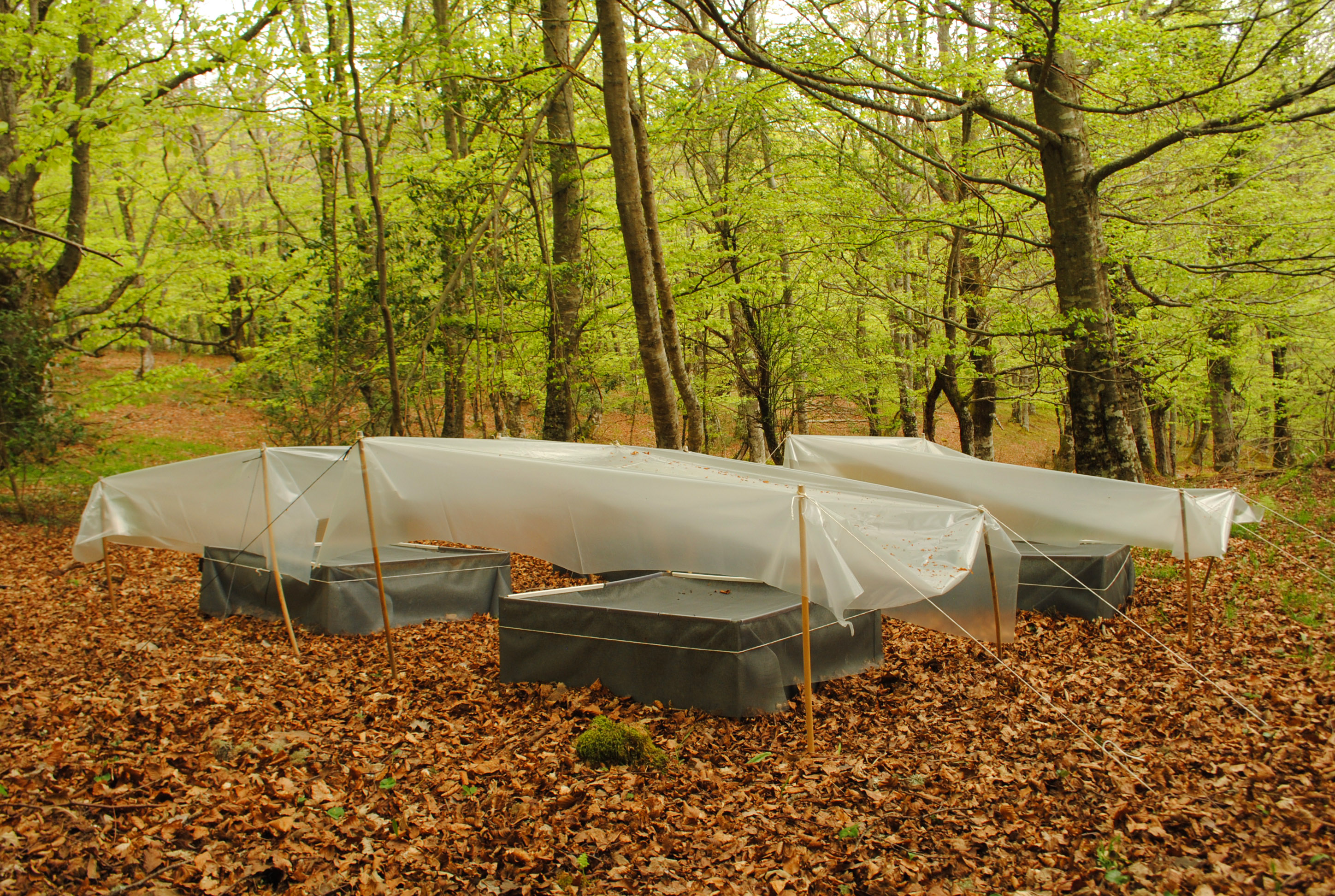
**

**Figure S2.** Short-term field experiment, showing the 1 m^2^ plots covered with plastic roofs used for rainfall exclusion. Photo credit: Nereida Melguizo-Ruiz.

**Supplemental  Information  - Appendix 2**

**Multiplicative effects of treatments on total abundances in the leaf litter (inside and outside the cupules)**


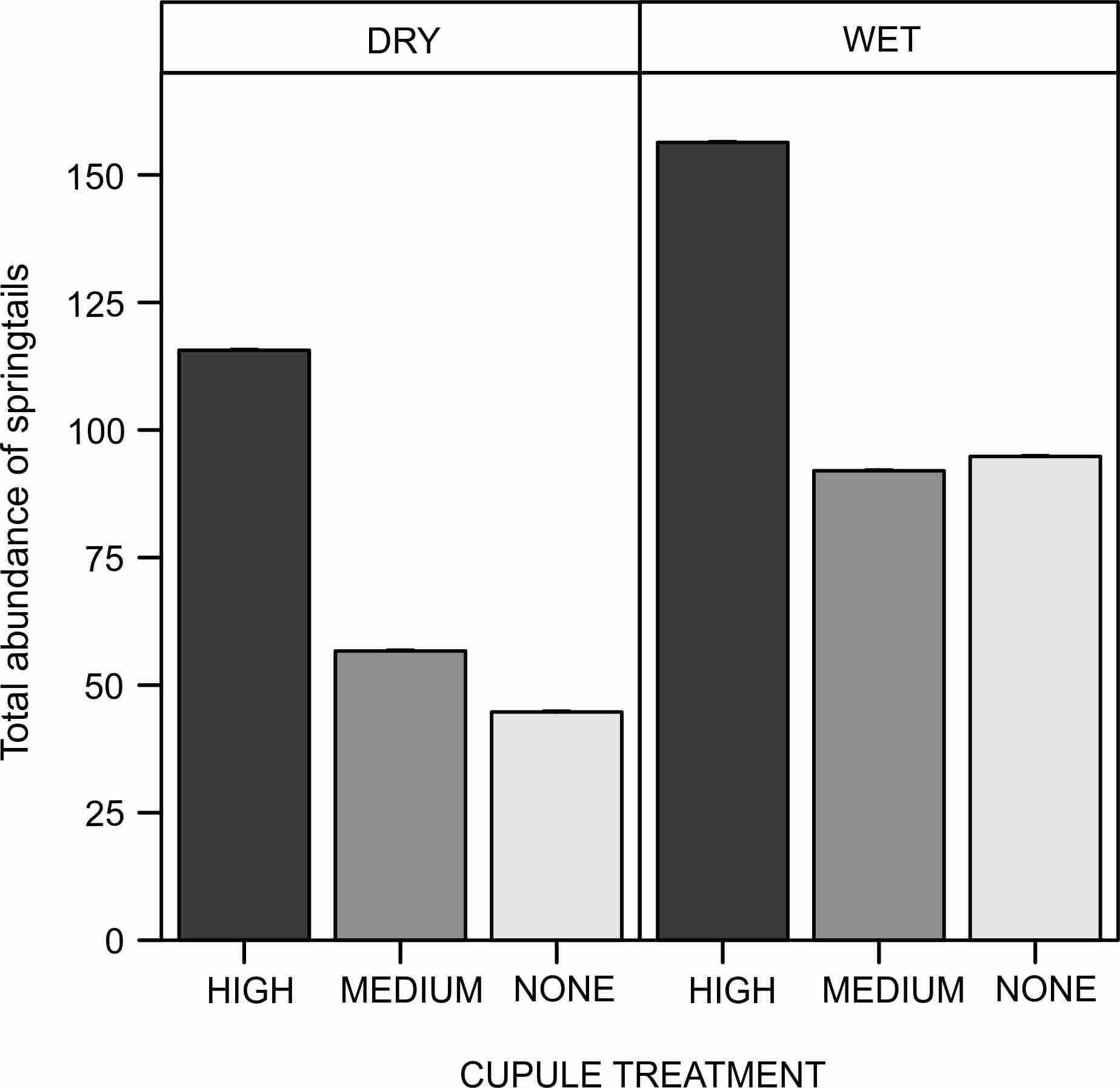


**Figure S3.** Total abundance of springtails in the leaf litter (inside and outside the cupules). Abbreviations: DRY: 'Dry' treatment, WET: 'Wet' treatment. HIGH: 'High density of cupules', MEDIUM: 'Intermediate density of cupules' and NONE: 'None' (i.e. no single cupule) treatment. Effects are model predicted effects ± SE (library "effects" – Fox 2003).

**Additive effects of treatments on the abundances inside relative to outside the cupules**

**
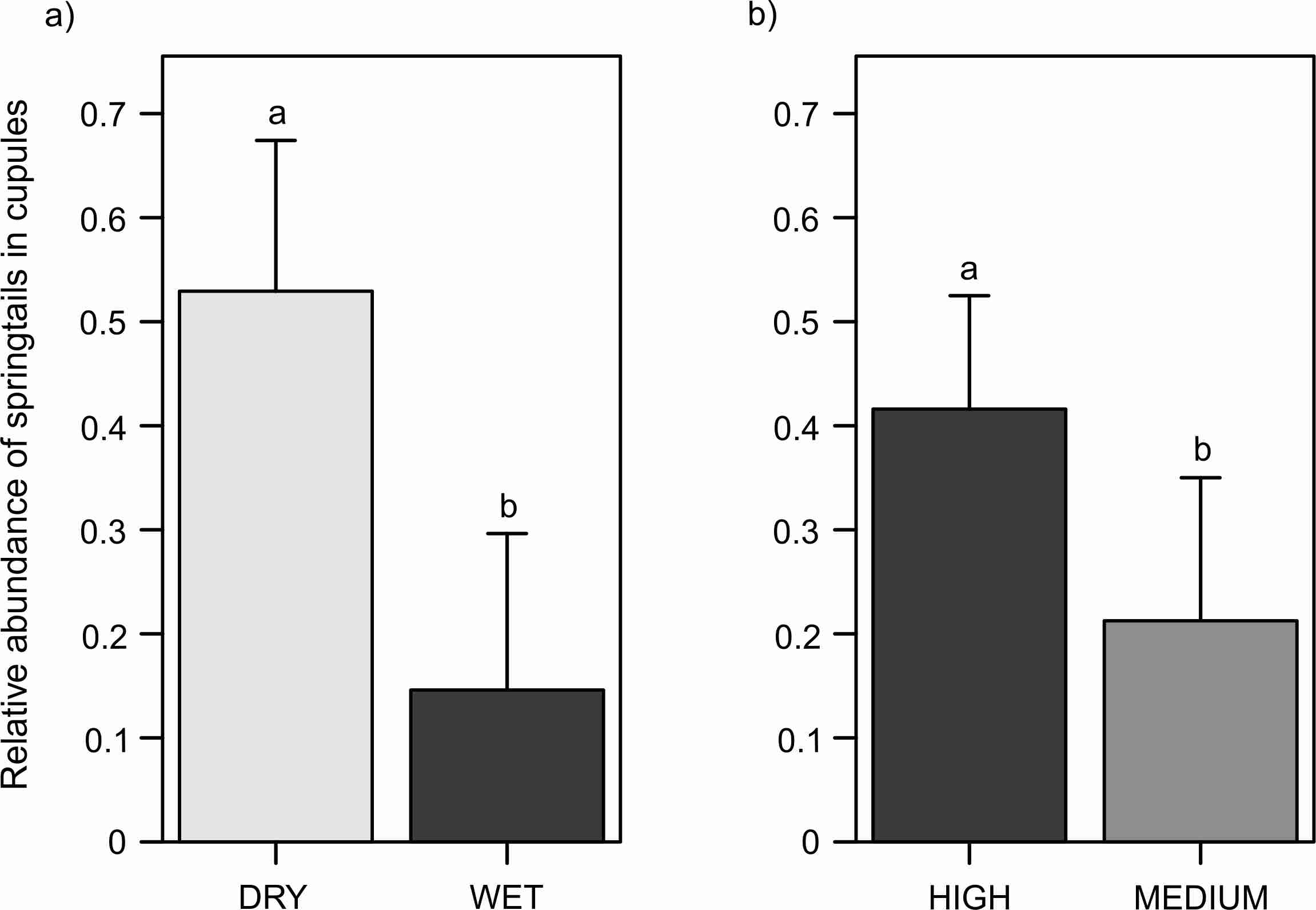
**

**Figure S4.** Abundance of springtails inside the cupules relative to outside, for a) water and b) cupule treatments. Abbreviations: DRY: 'Dry' treatment, WET: 'Wet' treatment. HIGH: 'High density of cupules' and MEDIUM: 'Intermediate density of cupules' treatment. Effects are model predicted effects ± SE (library "effects" – Fox 2003). Letters denote significant differences between treatments.

**
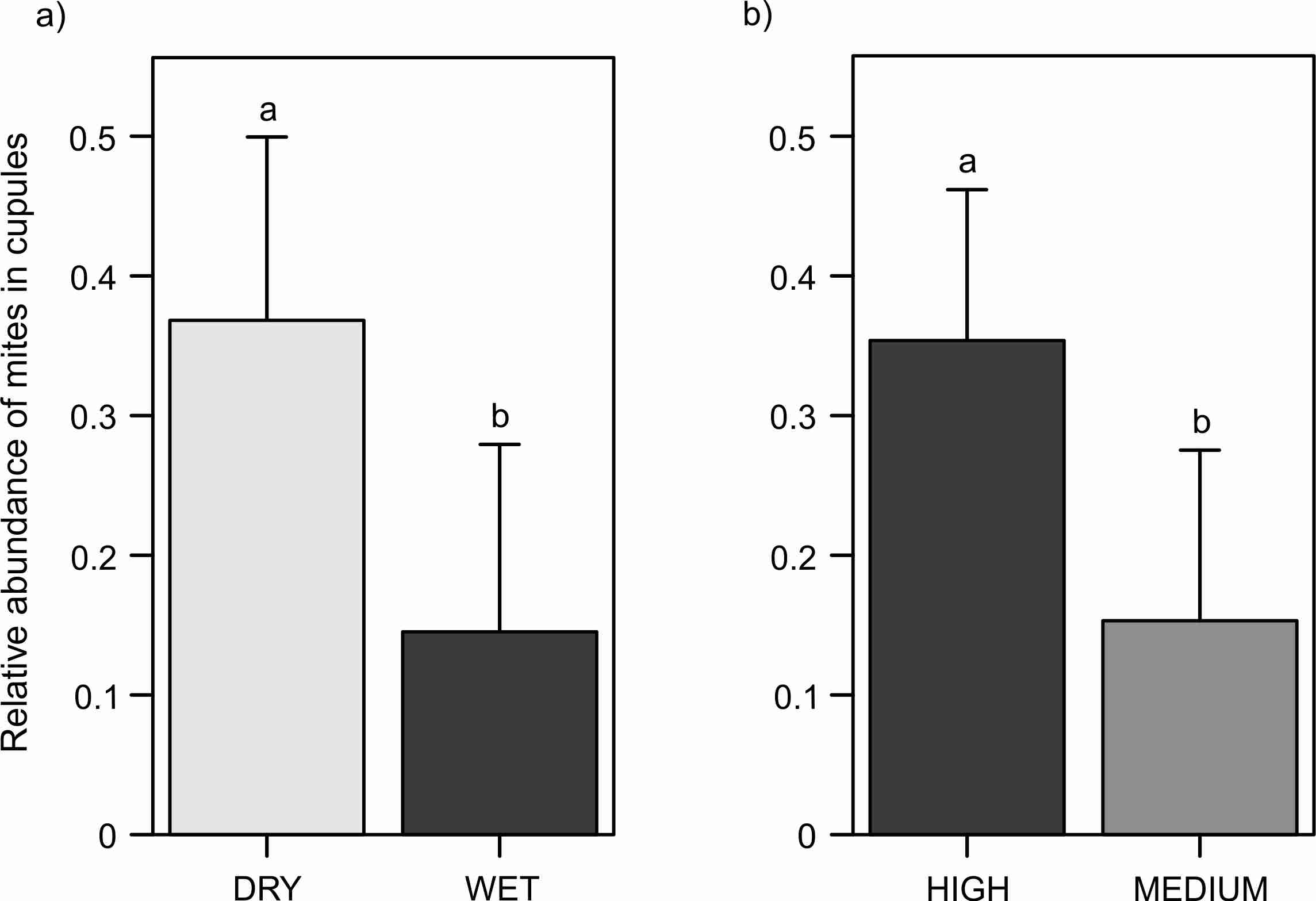
**

**Figure S5.** Abundance of mites inside the cupules relative to outside, for a) water and b) cupule treatments. Abbreviations: DRY: 'Dry' treatment, WET: 'Wet' treatment. HIGH: 'High density of cupules' and MEDIUM: 'Intermediate density of cupules' treatment. Effects are model predicted effects ± SE (library "effects" – Fox 2003). Letters denote significant differences between treatments.


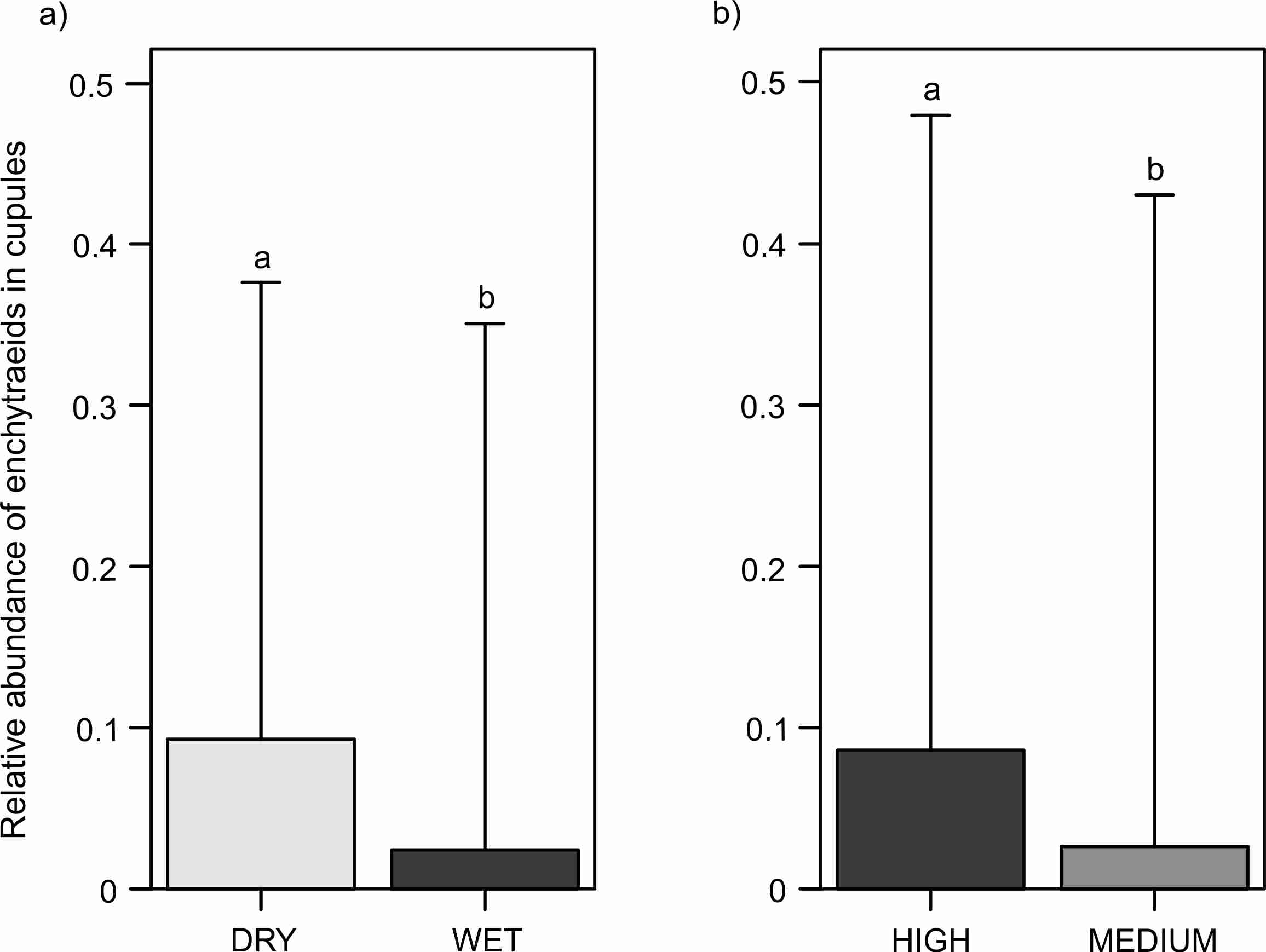


**Figure S6.** Abundance of enchytraeids inside the cupules relative to outside, for a) water and b) cupule treatments. Abbreviations: DRY: 'Dry' treatment, WET: 'Wet' treatment. HIGH: 'High density of cupules' and MEDIUM: 'Intermediate density of cupules' treatment. Effects are model predicted effects ± SE (library "effects" – Fox 2003). Letters denote significant differences between treatments.

**Additive effects of treatments on total abundances in the leaf litter (inside and outside the cupules)**


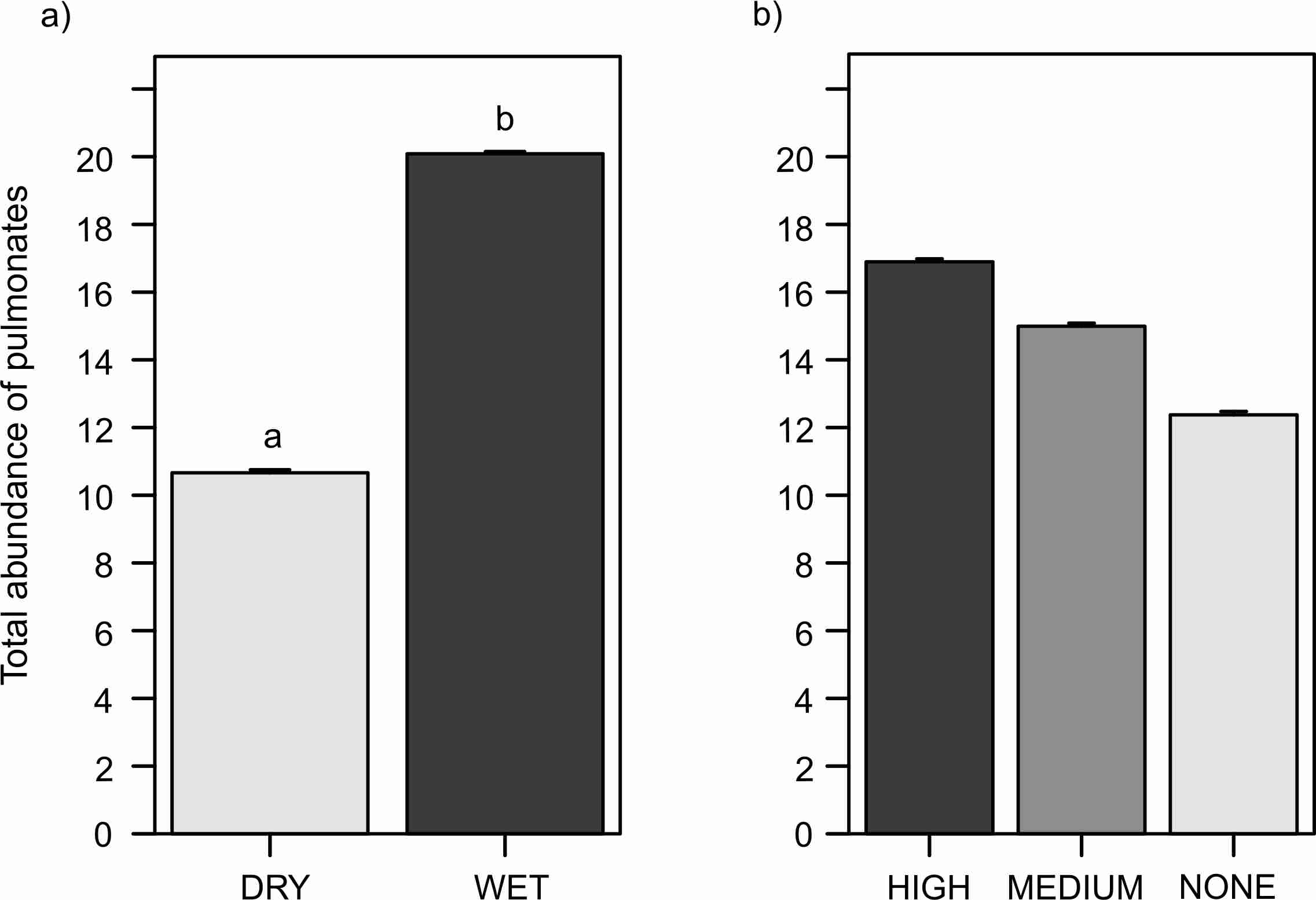


**Figure S7.** Total abundance of pulmonates in the leaf litter (inside and outside the cupules), across a) water and b) cupule treatments. Abbreviations: DRY: 'Dry' treatment, WET: 'Wet' treatment. HIGH: 'High density of cupules', MEDIUM: 'Intermediate density of cupules' and NONE: 'None' (i.e. no single cupule) treatment. Effects are model predicted effects ± SE (library "effects" – Fox 2003). Letters denote significant differences between treatments after post-hoc contrasts.


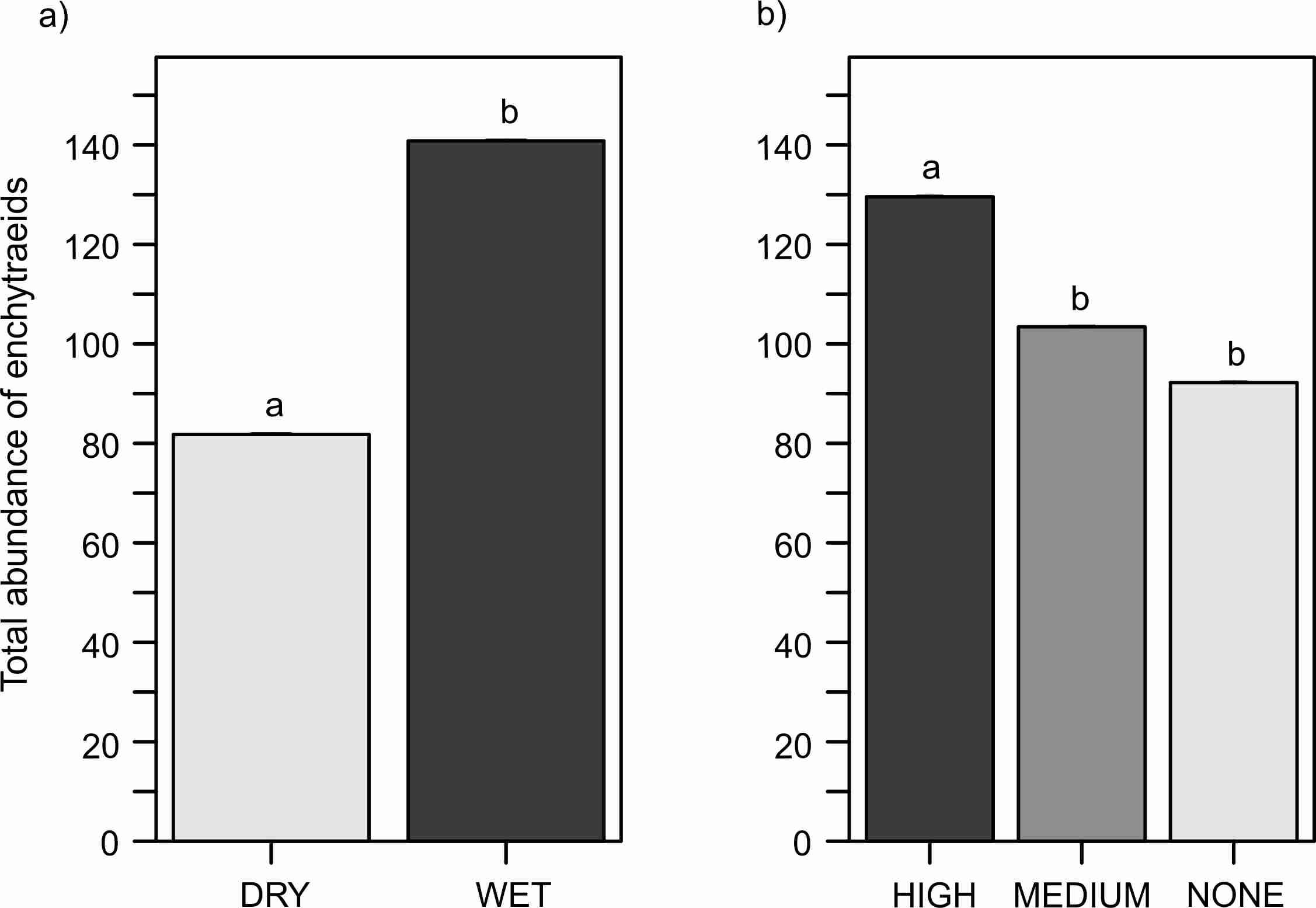


**Figure S8.** Total abundance of enchytraeids in the leaf litter (inside and outside the cupules), across a) water and b) cupule treatments. Abbreviations: DRY: 'Dry' treatment, WET: 'Wet' treatment. HIGH: 'High density of cupules', MEDIUM: 'Intermediate density of cupules' and NONE: 'None' (i.e. no single cupule) treatment. Effects are model predicted effects ± SE (library "effects" – Fox 2003). Letters denote significant differences between treatments after post-hoc contrasts.

**
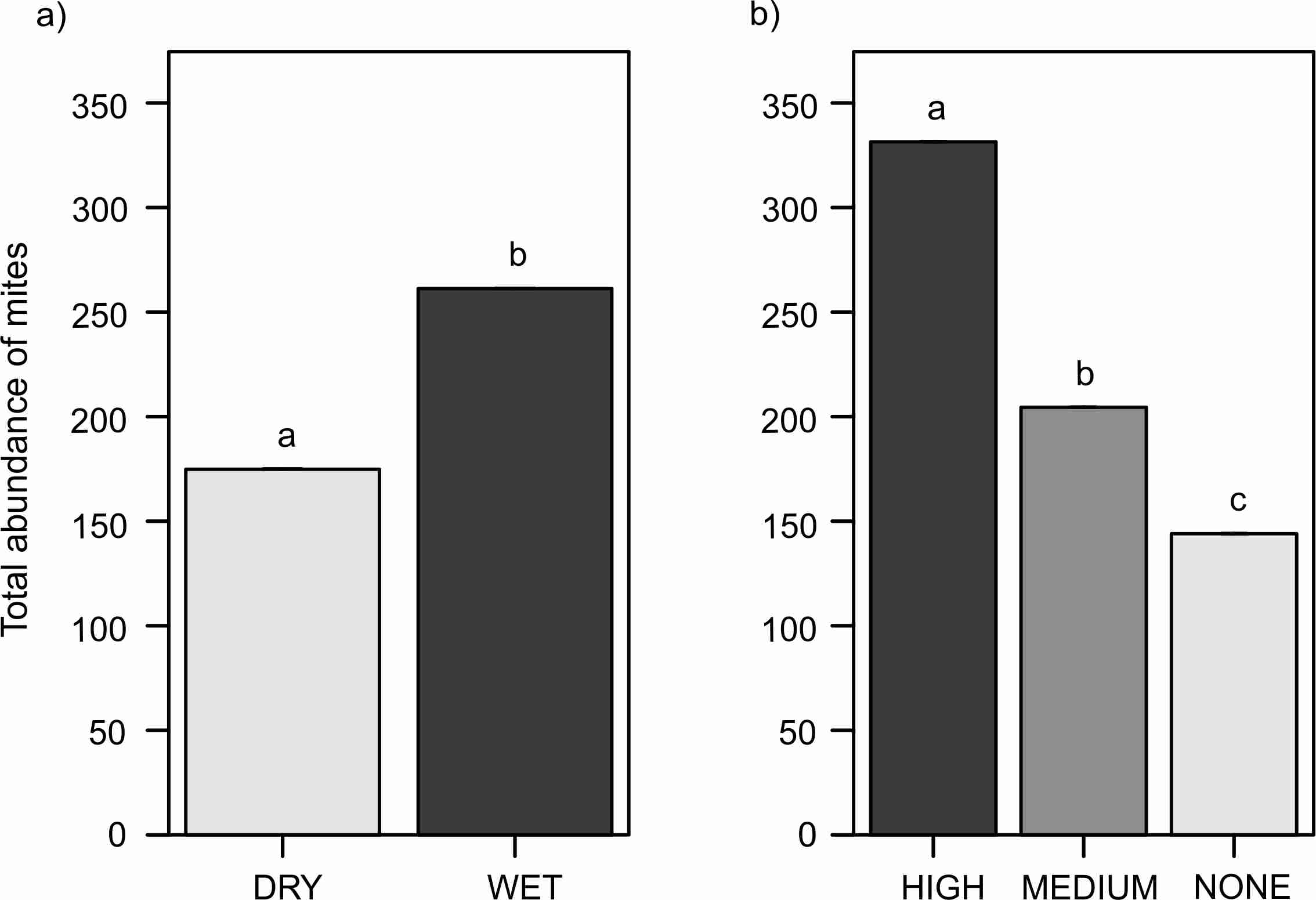
**

**Figure S9.** Total abundance of mites in the leaf litter (inside and outside the cupules), across a) water and b) cupule treatments. Abbreviations: DRY: 'Dry' treatment, WET: 'Wet' treatment. HIGH: 'High density of cupules', MEDIUM: 'Intermediate density of cupules' and NONE: 'None' (i.e. no single cupule) treatment. Effects are model predicted effects ± SE (library "effects" – Fox 2003). Letters denote significant differences between treatments after post-hoc contrasts.

**Supplemental  Information  - Appendix 3**

**Table S1.** Total abundances of fauna in the leaf litter, inside and outside the cupules (Mean ± sd).

|  | **WATER TREATMENT** | | **CUPULE TREATMENT** | | |
| --- | --- | --- | --- | --- | --- |
| **TAXONOMIC GROUPS** | WET | DRY | HIGH | INTERMEDIATE | NO CUPULES |
|  |  |  | DENSITY | DENSITY |  |
|  | Mean ± SD | Mean ± SD | Mean ± SD | Mean ± SD | Mean ± SD |
| **ARACHNIDA** |  |  |  |  |  |
| ARANEAE | 4.1 ± 2.6 | 3.4 ± 2.3 | 3.3 ± 2.2 | 3.5 ± 2.6 | 4.5 ±2.7 |
| **ACARINA (TOTAL)** | 282.2 ± 116.3 | 189.8 ± 84.7 | 344.5 ± 100.9 | 212.3 ± 61.6 | 151.1 ± 59.7 |
| ORIBATIDA | 177.1 ± 75.4 | 104.8 ± 44.7 | 206.0 ± 69.9 | 129.1 ± 42.5 | 87.8 ± 40.4 |
| MESOSTIGMATA | 95.2 ± 41.1 | 76.9 ± 42.8 | 126.9 ± 42.4 | 73.6 ±22.6 | 57.6 ± 22.3 |
| PROSTIGMATA | 9.6 ± 3.7 | 7.9 ± 4.4 | 11.4 ± 4.1 | 9.3 ± 1.9 | 5.6 ± 3.9 |
| PSEUDOSCORPIONIDA | 9.8 ± 4.4 | 8.0 ± 4.4 | 11.0 ± 4.1 | 7.9 ± 4.4 | 7.8 ± 4.3 |
| OPILIONES | 1.2 ± 1.3 | 0.3 ± 0.5 | 0.9 ± 1.4 | 0.8 ± 1.0 | 0.5 ± 0.8 |
| **MYRIAPODA** |  |  |  |  |  |
| LITHOBIOMORPHA | 5.1 ± 2.8 | 3.3 ± 2.9 | 5.4 ± 3.7 | 2.3 ± 0.9 | 4.9 ± 2.9 |
| GEOPHILOMORPHA | 4.9 ± 3.5 | 3.2 ± 2.0 | 4.5 ± 3.3 | 3.5 ± 3.3 | 4.1 ± 2.4 |
| JULIDA | 1.3 ± 1.1 | 0.8 ± 1.2 | 1.1 ± 1.4 | 1.1 ± 1.1 | 0.9 ± 1.0 |
| GLOMERIDA | NA | NA | NA | NA | NA |
| POLYXENIDA | 1.3 ± 1.5 | 0.7 ± 0.8 | 0.8 ± 0.7 | 1.3 ± 1.8 | 0.9 ± 1.1 |
| SYMPHYLA | 1.1 ± 1.0 | 0.8 ± 1.2 | 0.9 ± 1.1 | 0.5 ± 0.8 | 1.4 ± 1.3 |
| **HEXAPODA** |  |  |  |  |  |
| **COLLEMBOLA (TOTAL)** | 117.0 ± 42.3 | 74.4 ± 40.7 | 139.1 ± 38.9 | 75.6 ± 23.7 | 72.4 ± 40.6 |
| ENTOMOBRYOMORPHA | 87.4 ± 28.7 | 62.1 ± 38.5 | 109.9 ± 27.0 | 60.5 ± 20.0 | 53.9 ± 30.0 |
| PODUROMORPHA | 28.8 ± 18.1 | 11.5 ± 5.7 | 28.5 ± 22.7 | 14.4 ± 4.0 | 17.6 ± 13.0 |
| CAMPODEIDAE | 7.8 ± 3.4 | 5.0 ± 3.4 | 4.9 ± 2.7 | 5.4 ± 2.8 | 9.0 ± 4.0 |
| STAPHYLINIDAE | NA | NA | NA | NA | NA |
| CARABIDAE | NA | NA | NA | NA | NA |
| LARVAE | 12.5 ± 6.1 | 9.8 ± 2.9 | 15.5 ± 4.5 | 9.9 ± 2.6 | 8.1 ± 4.1 |
| **CRUSTACEA** |  |  |  |  |  |
| ISOPODA | 0.5 ± 0.7 | 0.6 ± 0.8 | 0.3 ± 0.5 | 0.8 ± 0.7 | 0.6 ± 0.9 |
| **ANNELIDA** |  |  |  |  |  |
| ENCHYTRAEIDAE | 146.5 ± 46.0 | 83.3 ± 16.0 | 137.6 ± 53.8 | 108.1 ± 32.8 | 98.9 ± 47.4 |
| LUMBRICIDAE | 2.9 ± 2.2 | 1.0 ± 1.3 | 2.8 ± 2.7 | 1.4 ± 1.2 | 1.8 ± 1.7 |
| **MOLLUSCA** |  |  |  |  |  |
| PULMONATA | 20.3 ± 5.2 | 10.8 ± 4.4 | 17.8 ± 8.8 | 15.8 ± 5.2 | 13.0 ± 5.7 |

**Table S2.** Statistical results of the a) multiplicative and b) additive GLMMs explaining total abundances of fauna in the leaf litter (inside and outside the cupules).

| a) |  |  |  |  |  |  |
| --- | --- | --- | --- | --- | --- | --- |
|  | **TAXONOMIC GROUPS** |  |  | χ² | df | p value |
|  | **COLLEMBOLA (TOTAL)** | WATER TREATMENT | | 2.9 | 1 | 0.0887 |
|  |  | CUPULE TREATMENT | | 77.4 | 2 | <0.0001 |
|  |  | **WATER*CUPULE** | | **8.4** | **2** | **0.0152** |
|  | ENTOMOBRYOMORPHA | WATER TREATMENT | | 0.4 | 1 | 0.5419 |
|  |  | CUPULE TREATMENT | | 108.3 | 2 | <0.0001 |
|  |  | **WATER*CUPULE** | | **16.8** | **2** | **0.0002** |

| b) |  | **WATER TREATMENT** | | | | | **CUPULE TREATMENT** | | | | | |
| --- | --- | --- | --- | --- | --- | --- | --- | --- | --- | --- | --- | --- |
|  | **TAXONOMIC GROUPS** | WET | DRY |  |  |  | HIGH | INTERMEDIATE | NO CUPULES |  |  |  |
|  |  |  |  |  |  |  | DENSITY | DENSITY |  |  |  |  |
|  |  | Estimate ± SE | Estimate ± SE | χ² | df | p value | Estimate ± SE | Estimate ± SE | Estimate ± SE | χ² | df | p value |
|  | **ARACHNIDA** |  |  |  |  |  |  |  |  |  |  |  |
|  | ARANEAE | 4.0 ± 0.1 | 3.4 ± 0.2 | 0.7 | 1 | 0.3997 | 3.2 ± 0.2 | 3.5 ± 0.2 | 4.5 ± 0.2 | 1.9 | 2 | 0.3959 |
|  | **ACARINA (TOTAL)** | **261.2 ± 0.1** | **174.8 ± 0.1** | **8.4** | **1** | **0.0039** | **331.4 ± 0.1** | **204.5 ± 0.1** | **144.0 ± 0.1** | **250.4** | **2** | **<0.0001** |
|  | ORIBATIDA | **162.9 ± 0.1** | **96.7 ± 0.1** | **14** | **1** | **0.0002** | **195.4 ± 0.1** | **122.8 ± 0.1** | **82.4 ± 0.1** | **169.2** | **2** | **<0.0001** |
|  | MESOSTIGMATA | 88.0 ± 0.1 | 68.6 ± 0.1 | 1.8 | 1 | 0.1809 | **121.1 ± 0.1** | **70.8 ± 0.1** | **54.8 ± 0.1** | **72.8** | **2** | **<0.0001** |
|  | PROSTIGMATA | 9.1 ± 0.1 | 7.5 ± 0.1 | 1.4 | 1 | 0.2451 | **11.2 ± 0.1** | **9.1 ± 0.1** | **5.6 ± 0.2** | **15** | **2** | **0.0006** |
|  | PSEUDOSCORPIONIDA | 9.2 ± 0.2 | 7.5 ± 0.2 | 0.7 | 1 | 0.3993 | 10.5 ± 0.2 | 7.5 ± 0.2 | 7.3 ± 0.2 | 5 | 2 | 0.0808 |
|  | OPILIONES | 0.9 ± 0.5 | 0.2 ± 0.7 | 3.5 | 1 | 0.0611 | 0.5 ± 0.5 | 0.5 ± 0.5 | 0.3 ± 0.6 | 0.8 | 2 | 0.6681 |
|  | **MYRIAPODA** |  |  |  |  |  |  |  |  |  |  |  |
|  | LITHOBIOMORPHA | 4.7 ± 0.2 | 3.0 ± 0.2 | 2.8 | 1 | 0.0953 | **5.2 ± 0.2** | **2.2 ± 0.3** | **4.7 ± 0.2** | **10.3** | **2** | **0.0059** |
|  | GEOPHILOMORPHA | 4.4 ± 0.2 | 2.9 ± 0.2 | 2.1 | 1 | 0.1437 | 4.0 ± 0.2 | 3.1 ± 0.3 | 3.7 ± 0.2 | 0.5 | 2 | 0.7655 |
|  | JULIDA | 1.2 ± 0.9 | 0.8 ± 0.6 | 0.7 | 1 | 0.4168 | 1.0 ± 0.4 | 1.0 ± 0.4 | 0.8 ± 0.4 | 0.3 | 2 | 0.8508 |
|  | GLOMERIDA | NA | NA | NA | NA | NA | NA | NA | NA | NA | NA | NA |
|  | POLYXENIDA | 1.0 ± 0.5 | 0.6 ± 0.6 | 0.5 | 1 | 0.4724 | 0.6 ± 0.5 | 1.0 ± 0.5 | 0.7 ± 0.5 | 1.1 | 2 | 0.5628 |
|  | SYMPHYLA | 0.8 ± 0.5 | 0.4 ± 0.7 | 0.8 | 1 | 0.377 | 0.6 ± 0.6 | 0.3 ± 0.6 | 0.9 ± 0.5 | 3.2 | 2 | 0.2059 |

|  | **WATER TREATMENT** | | | | | **CUPULE TREATMENT** | | | | | |
| --- | --- | --- | --- | --- | --- | --- | --- | --- | --- | --- | --- |
| **TAXONOMIC GROUPS** | WET | DRY |  |  |  | HIGH | INTERMEDIATE | NO CUPULES |  |  |  |
|  |  |  |  |  |  | DENSITY | DENSITY |  |  |  |  |
|  | Estimate ± SE | Estimate ± SE | χ² | df | p value | Estimate ± SE | Estimate ± SE | Estimate ± SE | χ² | df | p value |
| **HEXAPODA** |  |  |  |  |  |  |  |  |  |  |  |
| **COLLEMBOLA (TOTAL)** | **110.1 ± 0.1** | **67.2 ± 0.1** | **9.9** | **1** | **0.0016** | **133.8 ± 0.1** | **72.2 ± 0.1** | **65.9 ± 0.1** | **68.6** | **2** | **<0.0001** |
| ENTOMOBRYOMORPHA | NA significant interaction (see above) | | | | | NA significant interaction (see above) | | | | | |
| PODUROMORPHA | **25.3 ± 0.2** | **10.6 ± 0.2** | **8.4** | **1** | **0.0038** | **22.7 ± 0.2** | **13.3 ± 0.2** | **14.6 ± 0.2** | **9.6** | **2** | **0.0082** |
| CAMPODEIDAE | 7.4 ± 0.2 | 4.6 ± 0.2 | 2.8 | 1 | 0.0971 | **4.6 ± 0.2** | **5.1 ± 0.2** | **8.5 ± 0.2** | **12.4** | **2** | **0.002** |
| STAPHYLINIDAE | NA | NA | NA | NA | NA | NA | NA | NA | NA | NA | NA |
| CARABIDAE | NA | NA | NA | NA | NA | NA | NA | NA | NA | NA | NA |
| LARVAE | 12.0 ± 0.1 | 9.4 ± 0.1 | 2.7 | 1 | 0.0991 | **15.3 ± 0.1** | **9.8 ± 0.1** | **8.0 ± 0.1** | **20.7** | **2** | **<0.0001** |
| **CRUSTACEA** |  |  |  |  |  |  |  |  |  |  |  |
| ISOPODA | 0.5 ± 0.4 | 0.5 ± 0.4 | 0.1 | 1 | 0.7817 | 0.2 ± 0.7 | 0.7 ± 0.4 | 0.6 ± 0.5 | 1.8 | 2 | 0.3992 |
| **ANNELIDA** |  |  |  |  |  |  |  |  |  |  |  |
| ENCHYTRAEIDAE | **140.8 ± 0.1** | **81.8 ± 0.1** | **17.4** | **1** | **<0.0001** | **129.6 ± 0.1** | **103.4 ± 0.1** | **92.2 ± 0.1** | **23.4** | **2** | **<0.0001** |
| LUMBRICIDAE | 2.6 ± 0.3 | 0.9 ± 0.4 | 5.8 | 1 | 0.0157 | 2.3 ± 0.3 | 1.1 ± 0.3 | 1.4 ± 0.3 | 4.1 | 2 | 0.1312 |
| **MOLLUSCA** |  |  |  |  |  |  |  |  |  |  |  |
| PULMONATA | **20.1 ± 0.1** | **10.7 ± 0.1** | **33.8** | **1** | **<0.0001** | 16.9 ± 0.1 | 15.0 ± 0.1 | 12.4 ± 0.1 | 5.8 | 2 | 0.0541 |
|  |  |  |  |  |  |  |  |  |  |  |  |
| ***** Significant p-values after applying the False Discovery Rate procedure are shown in bold | | | | | | | |  |  |  |  |
| Estimates (±SE) are the predicted means of each factor extracted from the GLMM models (library "effects") | | | | | | | |  |  |  |  |
| NA - Insoluble models -likely due to the small number of individuals present- | | | | | |  |  |  |  |  |  |
